# Thyroid Dysfunction and Associated Risk Factors among Nepalese Diabetes Mellitus Patients

**DOI:** 10.1155/2015/570198

**Published:** 2015-09-07

**Authors:** Saroj Khatiwada, Rajendra KC, Santosh Kumar Sah, Seraj Ahmed Khan, Rajendra Kumar Chaudhari, Nirmal Baral, Madhab Lamsal

**Affiliations:** ^1^Department of Pharmacy, Central Institute of Science and Technology (CIST) College, Pokhara University, Kathmandu, Nepal; ^2^Department of Medical Laboratory Technology, Modern Technical College, Satdobato, Lalitpur, Nepal; ^3^Department of Biochemistry, Universal College of Medical Sciences, Bhairahawa, Nepal; ^4^Department of Biochemistry, B. P. Koirala Institute of Health Sciences, Dharan, Nepal

## Abstract

*Objectives*. To assess thyroid function and associated risk factors in Nepalese diabetes mellitus patients. *Methods*. A cross-sectional study was carried out among 419 diabetes mellitus patients at B. P. Koirala Institute of Health Sciences, Dharan, Nepal. Information on demographic and anthropometric variables and risk factors for thyroid dysfunction was collected. Blood samples were analysed to measure thyroid hormones, blood sugar, and lipid profile. *Results*. Prevalence rate of thyroid dysfunction was 36.03%, with subclinical hypothyroidism (26.5%) as the most common thyroid dysfunction. Thyroid dysfunction was much common in females (42.85%) compared to males (30.04%) (*p* = 0.008) and in type 1 diabetes (50%) compared to type 2 diabetes mellitus (35.41%) (*p* = 0.218). Diabetic patients with thyroid dysfunction had higher total cholesterol, HDL cholesterol, and LDL cholesterol in comparison to patients without thyroid dysfunction. Significant risk factors for thyroid dysfunction, specifically hypothyroidism (overt and subclinical), were smoking (relative risk of 2.56 with 95% CI (1.99–3.29, *p* < 0.001)), family history of thyroid disease (relative risk of 2.57 with 95% CI (2.0–3.31, *p* < 0.001)), and female gender (relative risk of 1.44 with 95% CI (1.09–1.91, *p* = 0.01)). *Conclusions*. Thyroid dysfunction is common among Nepalese diabetic patients. Smoking, family history of thyroid disease, and female gender are significantly associated with thyroid dysfunction.

## 1. Introduction

Diabetes mellitus is being one of the greatest health threats for the 21st century [1]. Prevalence of diabetes is rising rapidly in developing countries, and the global number of diabetes is estimated to reach 366 million in 2030 among adults aged ≥20 years [1, 2]. Both type 1 and type 2 diabetes are powerful and independent risk factors for coronary artery disease (CAD), stroke, and peripheral arterial disease [3]. The global rise in diabetes burden has led to significant increase in health care expenditure [2].

There is a deep underlying relation between diabetes mellitus and thyroid dysfunction [4]. Studies have found that thyroid dysfunction is much common in diabetic population compared to nondiabetic population, and diabetes and thyroid disorders have been shown to mutually influence each other [5, 6]. Most often thyroid dysfunction and type 1 diabetes are due to an autoimmune condition, whereas type 2 diabetes is mainly due to insulin resistance [7, 8]. Thus, it seems that diabetes, especially type 1, has potential link with thyroid dysfunction or vice versa. Besides the effects due to high blood glucose in diabetics, low thyroid hormones independently increase the risk for cardiovascular diseases in both diabetic and nondiabetic patients [3, 9].

Latest reports suggest that diabetes is rising rapidly particularly in developing countries from Asia [2]. A study in eastern Nepal reported diabetes in 6.3% population [10]. Hence, assessment of thyroid function in the uprising diabetic patient number may be helpful in identifying cases of clinical and subclinical thyroid dysfunction thereby assisting in mitigating the harmful effects due to low thyroid hormones. Thyroid profile in Nepalese population with diabetes mellitus has not been studied earlier. Thus, we designed a study by selecting confirmed diabetic patients from eastern Nepal and performed clinical and laboratory evaluation to investigate thyroid function status and associated risk factors.

## 2. Materials and Methods

A cross-sectional study was carried out among the diabetes mellitus patients attending biochemistry laboratory of B. P. Koirala Institute of Health Sciences, Dharan, Nepal, from February 2013 to January 2014. A total of 419 patients with diabetes mellitus were selected during the study period based on our inclusion criteria of having diabetes for at least 6 months. Those diabetic patients with known thyroid disorders, complications of diabetes mellitus, history of other illnesses, and hyperlipidemia as well as on corticosteroids therapy and on medications affecting thyroid function were excluded from the study. Diabetes mellitus was diagnosed on the basis of American Diabetes Association (ADA) criteria and classification of type 1 diabetes from type 2 diabetes was done on basis of physician classifications based on the age of onset of diabetes and dependence on insulin therapy alone to achieve normal plasma glucose concentrations [8]. Consent was taken from each patient and the study protocol was approved by the institute review board of B. P. Koirala Institute of Health Sciences, Dharan.

Each subject demographic (age, sex) and anthropometric measurements (height, weight, and BMI) including blood pressure (systolic and diastolic), duration of diabetes, family history of diabetes mellitus and thyroid disease, and alcohol intake habit and smoking habit was recorded. About 5 mL overnight fasting venous blood samples were collected at morning time from each patient. Blood glucose, glycated hemoglobin (HbA1c), total cholesterol, high density lipoprotein (HDL) cholesterol, low density lipoprotein (LDL) cholesterol, triglyceride, free triiodothyronine (T3), free tetraiodothyronine (T4), and thyroid stimulating hormone (TSH) were estimated. Blood glucose and HbA1c were estimated using enzymatic method and by Nycocard reader II based on immunochromatographic principle, respectively. Serum free T3, free T4, and TSH were measured by using fluorescent immunoassay (VIDAS, biomeriux SA, France). Similarly, total cholesterol and triglycerides were measured by enzymatic method (kits from AGAPPE diagnostics by Biolyzer 100). LDL cholesterol and HDL cholesterol were estimated by homogeneous, direct method (kits from Gesan by Biolyzer 100).

Thyroid dysfunction was said to occur if patients thyroid hormones fall outside the reference range (free T3 (4.0–8.3 pmol/L), free T4 (9.0–20.0 pmol/L), and TSH level (0.25–5 mIU/L)). The data generated from study was entered into MS excel and analyzed using SPSS version 11.0. Continuous variables were expressed as mean ± SD values and categorical variables were expressed as percentage (number). One-way ANOVA test was applied for continuous variables and chi square test was applied for categorical variables at 95% confidence interval. Relative risk with 95% confidence interval (CI) was used to assess various risk factors for thyroid dysfunction.

## 3. Results

The study population comprised 53.22% males and 46.77% females with mean age of 51.27 ± 15.33 years. The mean age of males and females was 50.93 ± 15.44 years and 51.66 ± 15.24 years, respectively. Type 1 and type 2 diabetes mellitus patients were 4.3% (*n* = 18) with mean age of 19.28 ± 1.07 years and 95.7% (*n* = 401) with mean age of 52.7 ± 14.05 years, respectively. The BMI among type 1 and type 2 diabetics was significantly different (22.64 ± 1.58 Kg/m^2^ versus 25.28 ± 2.81 Kg/m^2^, *p* < 0.001).

Euthyroidism, subclinical hypothyroidism, overt hypothyroidism, and subclinical hyperthyroidism among study population were 64% (*n* = 268), 26.5% (*n* = 111), 5.5% (*n* = 23) and 4.1% (*n* = 17), respectively. Significant difference in thyroid function status was observed among genders (*p* = 0.024). Significantly higher number of diabetic patients without thyroid dysfunction (*n* = 39, 9.3%) had hypertension history compared to those with hypothyroidism (both overt and subclinical) (*n* = 33, 7.87%) and subclinical hyperthyroidism (*n* = 3, 0.71%) (*p* = 0.046). Parameters of lipid profile were not significantly different among diabetic patients with thyroid dysfunction (overt and subclinical hypothyroidism and subclinical hyperthyroidism) and without thyroid dysfunction (euthyroidism). Free T3 (*p* < 0.001), free T4 (*p* < 0.001), and TSH (*p* < 0.001) were significantly different among diabetic patients with and without thyroid dysfunction. Demographic, anthropometric, and clinical parameters in the study population according to thyroid status, hypothyroidism (both overt and subclinical), subclinical hyperthyroidism, and euthyroidism are shown in Table 1. Similarly, the level of different biochemical parameters in the study population according to thyroid function status is shown in Table 2.

Diabetic males had significantly higher BMI (25.66 ± 2.8 Kg/m^2^ versus 24.59 ± 2.73 Kg/m^2^, *p* < 0.001), systolic blood pressure (121.84 ± 7.14 mmHg versus 119.72 ± 6.45 mmHg, *p* = 0.001), and fasting blood glucose (179.99 ± 54.72 mg/dL versus 172.5 ± 60.44 mg/dL, *p* = 0.005) than diabetic females, respectively. However, diabetic females had significantly higher HDL cholesterol (41.2 ± 4.17 mg/dL versus 39.46 ± 2.78 mg/dL, *p* < 0.001) and triglycerides (191.38 ± 105.82 mg/dL versus 172.32 ± 101.02 mg/dL, *p* = 0.024) than diabetic males. Diabetic males and females had no significant difference in diastolic blood pressure (81.41 ± 5.66 mmHg versus 80.45 ± 6.26 mmHg, *p* = 0.124), HbA1c (7.23 ± 1.99% versus 7.19 ± 1.75%, *p* = 0.411), total cholesterol (188.44 ± 54.91 mg/dL versus 192.44 ± 50.97 mg/dL, *p* = 0.187), LDL cholesterol (112.11 ± 47.61 mg/dL versus 110.77 ± 41.72 mg/dL, *p* = 0.704), free T3 (5.03 ± 1.21 pmol/L versus 5.04 ± 1.39 pmol/L, *p* = 0.587), free T4 (11.78 ± 3.46 pmol/L versus 11.78 ± 3.61 pmol/L, *p* = 0.914), and TSH (4.02 ± 3.27 mIU/L versus 5.29 ± 5.03 mIU/L, *p* = 0.135), respectively.

Thyroid dysfunction was more common in type 1 diabetic patients (50%, *n* = 9) as compared to type 2 diabetic patients (35.41%, *n* = 142) and in diabetic females (42.85%, *n* = 84) than in diabetic males (30.04%, *n* = 67). Hypothyroidism was the only thyroid dysfunction in type 1 diabetes patients, whereas it was the major thyroid dysfunction in type 2 diabetes patients (31.17%, *n* = 125). Significant risk factors for thyroid dysfunction were smoking (relative risk 2.32 with 95% CI (1.85–2.91, *p* < 0.001)), family history of thyroid disease (relative risk 2.31 with 95% CI (1.84–2.91, *p* < 0.001)), and female gender (relative risk 1.42 with 95% CI (1.1–1.84, *p* = 0.006)). When separately analyzed, significant risk factors for hypothyroidism included smoking (relative risk of 2.56 with 95% CI (1.99–3.29, *p* < 0.001)), family history of thyroid disease (relative risk of 2.57 with 95% CI (2.0–3.31, *p* < 0.001)), and female gender (relative risk of 1.44 with 95% CI (1.09–1.91, *p* = 0.01)). However, smoking, family history of thyroid disease, and female gender did not appear as significant risk factors for subclinical hyperthyroidism. Age ≥60 years and duration of diabetes ≥5 years had relative risk of 1.03 with 95% CI (0.79–1.35, *p* = 0.79) and 1.3 with 95% CI (1.0–1.68, *p* = 0.053), respectively, for thyroid dysfunction.

## 4. Discussion

Diabetic patients have susceptibility to different types of thyroid dysfunction, whether hypothyroidism or hyperthyroidism; at the same time, patients with thyroid dysfunction are susceptible to suffer from either type 1 diabetes or type 2 diabetes [5]. The present study finds thyroid dysfunction as a common endocrine disorder in diabetic patients, where we reported that 36.03% diabetic patients had thyroid dysfunction. Studies have reported high prevalence of thyroid dysfunction in Nepal even in the general population. A hospital based study by Baral et al. in eastern Nepal reported hypothyroidism and hyperthyroidism in 17.19% and 13.68% population, respectively [11]. Similarly, in a study in Kavre district of central Nepal, thyroid dysfunction was observed in 31.84% metabolic syndrome patients [12]. While iodine nutrition has been rapidly improving in the past years in Nepal, excess iodine intake, as indicated by recent studies in school children of eastern Nepal, could also be a responsible factor for the high prevalence of thyroid dysfunction specifically hypothyroidism in our study population. Both iodine excess and deficiency have been found to affect thyroid gland function [13]. The most common thyroid dysfunction in the present study was subclinical hypothyroidism (26.5%) followed by overt hypothyroidism (5.5%) and subclinical hyperthyroidism (4.1%). We reported higher prevalence of thyroid dysfunction in females (42.85%) than in males (30.04%), which has been well observed in many studies. Our results are in agreement with previous studies in India, Saudi Arabia, and Greece [5, 14, 15]. A retrospective study in India reported thyroid dysfunction in 31.2% of type 2 diabetic patients with 27.7% having hypothyroidism [14]. Similarly, a study in Saudi Arabia found thyroid dysfunction in 28.5% of type 2 diabetic patients, with 25.3% having hypothyroidism [5]. Prevalence of thyroid dysfunction was much lower than in our study among the type 2 diabetic patients of Greece where thyroid dysfunction prevalence was 12.3% (5.53% in males and 18.48% in females) [15].

We observed that thyroid dysfunction is more common in type 1 diabetic patients (50%) than in type 2 diabetic patients (35.41%). Various studies have revealed that autoimmune thyroid disease is the commonest autoimmune disorder associated with type 1 diabetes. Type 1 diabetes and autoimmune thyroid disease share an autoimmune disposition, and recent studies have shown a shared genetic susceptibility to both conditions [16]. Kordonouri et al. also found thyroid dysfunction to be more common in subjects with type 1 diabetes compared to those with type 2 diabetes and a 3.5-fold increased risk of autoimmune thyroiditis was noticed in GADA positive patients [17].

Thyroid hormones have prominent roles in regulating a number of metabolic pathways including lipoprotein metabolism [9]. We found that diabetic patients with thyroid disorders, hypothyroidism, or subclinical hyperthyroidism had higher total cholesterol and LDL cholesterol than those without thyroid dysfunction. Dyslipidemia is a reported complication of hypothyroidism in patients with or without diabetes. However, in the study in Greece, patients with thyroid dysfunction had higher HDL cholesterol levels and lower values of LDL cholesterol levels in comparison with patients without thyroid dysfunction [15].

The present study reveals that hypertension (17.9%), smoking habit (19.3%), alcohol intake (31.5%), family history of diabetes mellitus (32.9%), and family history of thyroid disease (20.8%) are common among Nepalese patients with diabetes. Among the various risk factors for thyroid dysfunction we studied, smoking, family history of thyroid disease, and female gender had significant risk for thyroid dysfunction, specifically hypothyroidism. Similar result was observed by Papazafiropoulou et al., who reported that presence of thyroid dysfunction was related with gender and LDL cholesterol levels in type 2 diabetic patients [15]. Similarly, Al-Geffari et al. found family history of thyroid disease, female gender, and duration of diabetes of >10 years as significant risk factor for thyroid dysfunction in type 2 diabetic patients [5]. In a study by Chubb et al. subclinical hypothyroidism was associated with anti-TPO status and age, but there were no independent associations with serum cholesterol, history of coronary heart disease, HbA1c, or hypoglycaemic therapy in diabetic women [18].

Thyroid disorders and diabetes mellitus have a complex interdependent interaction. In diabetic patients, the nocturnal TSH peak is blunted or abolished, and the TSH response to TRH is impaired. Reduced T3 levels have been observed in uncontrolled diabetic patients, which is due to impairment in peripheral conversion of T4 to T3 that normalizes with improvement in glycemic control. Higher levels of circulating insulin associated with insulin resistance have shown a proliferative effect on thyroid tissue resulting in larger thyroid size with increased formation of nodules [6]. Diabetic patients have strong chance of developing thyroid dysfunction over the time course. A study by Chubb et al., in women with type 2 diabetes without known thyroid disease at baseline, revealed that diabetic women develop subclinical hypothyroidism (8.6%) over time as found after 5 years [18]. Furthermore, it seems that unidentified thyroid dysfunction could negatively impact diabetes and its complications. A higher frequency of retinopathy and nephropathy was observed in diabetic patients with subclinical hypothyroidism, and more severe retinopathy was noted as well. Therefore, management of subclinical hypothyroidism in patients with diabetes may prove highly beneficial [6].

Thyroid function test is not a commonly recommended investigation in patients with diabetes especially in undeveloped countries like Nepal. Based on our findings and the reported harmful effects of low thyroid hormones on cardiovascular health, we recommend the frequent screening (1-2 years) of thyroid function in patients with diabetes. Our findings provide an evidence for the importance and necessity of thyroid function screening in patients with diabetes. This will assist in better clinical management of diabetes patients. The present study has however few limitations. The prevalence of anti-TPO antibodies in the study population could not be assessed. Assessment of anti-TPO antibodies would have provided clues for the thyroid autoimmunity status in the study population, which could have helped to explain the high rate of thyroid dysfunction. Autoimmunity against the thyroid gland is one of the most important causes for thyroid dysfunction. Anti-TPO antibody estimation helps in establishing the etiological diagnosis of autoimmune thyroid diseases. Anti-TPO antibodies are the most prevalent and present in 80–90% of Hashimoto's thyroiditis, 65–75% of grave's disease, and 10–20% of nodular goiter or thyroid carcinoma [19]. Another limitation of the present study was that thyroid function was tested only once, which may have resulted in the inclusion of subjects with nonthyroidal illness. The other limitation of the present study was that the iodine status of the study population was not assessed. Iodine excess and deficiency both are found to be associated with thyroid dysfunction and thus the concurrent prevalence of such iodine nutrition conditions in the study population may have some contributions to the high prevalence of thyroid dysfunction [20].

In conclusion, the present study identifies thyroid dysfunction, as well as prominently subclinical hypothyroidism, as a common disorder in Nepalese patients with diabetes. Diabetic patients with thyroid dysfunction had higher lipid levels (total cholesterol and LDL cholesterol) than patients without thyroid dysfunction, so diabetic patients with thyroid dysfunction are at higher risk for cardiovascular diseases than those with normal thyroid function. Similarly, smoking, family history of thyroid disease, and female gender were associated with thyroid dysfunction (mainly hypothyroidism) in the study population. Thus, frequent screening for thyroid dysfunction especially in diabetic patients with family history of thyroid disease, female gender, and smoking habit needs to be done.

## Figures and Tables

**Table 1:** General characteristics of the study population.

Variables	All patients (*n* = 419)	Diabetic patients with thyroid dysfunction (*n* = 151)		Diabetic patients without thyroid dysfunction (*n* = 268)	*p* value
		Hypothyroidism^*∗*^ (*n* = 134)	Subclinical hyperthyroidism (*n* = 17)		
Gender					
Male	53.22% (*n* = 223)	14.08% (*n* = 59)	1.9% (*n* = 8)	37.23% (*n* = 156)	0.024
Female	46.77% (*n* = 196)	17.89% (*n* = 75)	2.14% (*n* = 9)	26.73% (*n* = 112)	
Age (years)	51.27 ± 15.33	48.72 ± 17.58	54.47 ± 12.3	52.35 ± 14.14	0.055
Diabetes type					
Type 1 DM	4.3% (*n* = 18)	2.14% (*n* = 9)	—	2.14% (*n* = 9)	0.197
Type 2 DM	95.7% (*n* = 401)	29.83% (*n* = 125)	4.05% (*n* = 17)	61.81% (*n* = 259)	
Diabetes duration (years)	3.77 ± 2.63	4.04 ± 3.1	3.77 ± 2.97	3.64 ± 2.33	0.354
Hypertension	17.9% (*n* = 75)	7.87% (*n* = 33)	0.71% (*n* = 3)	9.3% (*n* = 39)	0.046
Smoking habit	19.3% (*n* = 81)	12.17% (*n* = 51)	0.71% (*n* = 3)	6.44% (*n* = 27)	<0.001
Alcoholism	31.5% (*n* = 132)	10.5% (*n* = 44)	0.95% (*n* = 4)	20.04% (*n* = 84)	0.736
Family history of diabetes mellitus	32.9% (*n* = 138)	10.73% (*n* = 45)	2.14% (*n* = 9)	20.04% (*n* = 84)	0.181
Family history of thyroid disease	20.8% (*n* = 87)	12.88% (*n* = 54)	0.71% (*n* = 3)	7.15% (*n* = 30)	<0.001
BMI (Kg/m^2^)	25.16 ± 2.82	24.98 ± 2.67	25.5 ± 2.62	25.23 ± 2.9	0.623
Systolic blood pressure (mmHg)	120.85 ± 6.9	121.37 ± 7.12	119.94 ± 6.3	120.65 ± 6.82	0.523
Diastolic blood pressure (mmHg)	80.96 ± 5.96	81.43 ± 6.19	80.24 ± 5.1	80.78 ± 5.9	0.52

The data is presented as percentage (number) and as mean ± SD. *p* value was calculated among diabetic subjects with hypothyroidism and hyperthyroidism and without thyroid dysfunction at 95% confidence interval. ^*∗*^Hypothyroidism group included both subclinical and overt hypothyroids.

**Table 2:** Biochemical parameters in the study population.

Variables	All patients (*n* = 419)	Diabetic patients with thyroid dysfunction (*n* = 151)		Diabetic patients without thyroid dysfunction (*n* = 268)	*p* value
		Hypothyroidism^*∗*^ (*n* = 134)	Subclinical hyperthyroidism (*n* = 17)		
Fasting blood glucose (mg/dL)	176.48 ± 57.53	174.13 ± 51.99	165.59 ± 53.25	178.35 ± 60.42	0.574
HbA1c %	7.21 ± 1.88	7.5 ± 2.13	6.73 ± 1.18	7.1 ± 1.77	0.076
Total cholesterol (mg/dL)	190.31 ± 53.08	191.05 ± 54.14	198.58 ± 46.13	189.41 ± 53.08	0.774
HDL cholesterol (mg/dL)	40.27 ± 3.6	40.72 ± 3.73	40.29 ± 3.01	40.05 ± 3.56	0.209
LDL cholesterol (mg/dL)	111.48 ± 44.9	112.09 ± 45.22	115.1 ± 45.21	110.95 ± 44.88	0.917
Triglycerides (mg/dL)	181.24 ± 103.61	178.72 ± 111.22	198.18 ± 99.52	181.42 ± 100.14	0.766
Free T3 (pmol/L)	5.03 ± 1.3	4.65 ± 1.21	5.63 ± 1.07	5.19 ± 1.31	<0.001
Free T4 (pmol/L)	11.78 ± 3.52	10.45 ± 2.54	13.75 ± 3.48	12.31 ± 3.75	<0.001
TSH (mIU/L)	4.62 ± 4.23	9.45 ± 4.31	0.18 ± 0.041	2.48 ± 1.12	<0.001

The data is presented as mean ± SD. *p* value was calculated among diabetic subjects with hypothyroidism and hyperthyroidism and without thyroid dysfunction at 95% confidence interval. ^*∗*^Hypothyroidism group included both subclinical and overt hypothyroids.
